# Numerical simulation of shoegear-rail coupling vibration under different initial contact forces

**DOI:** 10.1038/s41598-024-57569-y

**Published:** 2024-03-22

**Authors:** Tong Xing, Peihuo Peng, Like Pan, Caizhi Yang, Fan He

**Affiliations:** 1 Standards & Metrology Research Institute, China Academy of Railway Sciences Corporation Limited, Beijing, 100015 China; 2 School of Science, Beijing University of Civil Engineering and Architecture, Beijing, 100044 China

**Keywords:** Shoegear-rail system, Initial contact force, Vibration, Numerical simulation, Mechanical engineering, Applied mathematics

## Abstract

As cities have grown, conductor rail power supplies have been widely used in the field of urban rail transit. In order to improve the running performance of trains and reduce the occurrence of accidents, it is necessary to understand the vibration of shoegear-rail system under different initial contact forces and explore the dynamic performance of shoegear-rail system. Therefore, according to the structure of shoegear-rail system, a coupling model of shoegear-rail system is established in this paper. On the basis of the model, the numerical simulation of the shoegear-rail system under different initial contact forces is carried out, and finally the vibration data of the shoegear-rail system under different initial contact forces are obtained. The results show that with the increase of initial contact force in the range of 70–160 N, the vibration amplitude of the electric shoegear and the fluctuation amplitude of the contact force increase, but the maximum absolute shear force value of the conductor rail decreases. It indicates that the lower initial contact force, the better the performance of shoegear-rail system.

## Introduction

Rail transit plays an increasingly important role in the development of the city. At present, rail transit trains mainly have two power supply modes: catenary power supply^1–3^ and conductor rail power supply^4,5^. Conductor rail power supply has been widely used in the field of urban rail transit due to its strong anti-electromagnetic interference, good conductive performance, convenient installation and adjustment, low cost, strong anti-bad weather ability and other advantages. However, as the train running speed is higher and higher, the contact force between the electric shoegear and conductor rail changes dramatically, the number of impact and dynamic vibration amplitude are obviously greater, which cause some problems such as the separation of the electric shoegear from the conductor rail, the instability of the current, the serious wear of the electric shoegear, so that the stability and quality of the dynamic current become one of the bottlenecks in the application of the conductor rail power supply mode in high-speed trains.

In the study on the power supply mode of conductor rail, Yang^6^ paid attention to the wear condition of the guide rail system, proposed two kinds of mechanical-empirical wear models, and compared the models. Chen^7^ developed an online detection system, which uses laser ranging and fiber optic hard spot sensing technology to dynamically detect the shoegear-rail system. However, with the increase of train speed, the change of shoegear-rail system is closely related to the dynamic performance of shoegear-rail system. In the study of the dynamic performance of shoegear-rail system, there are roughly two research methods^8^, namely shoegear-rail dynamic simulation^9,10^ and shoegear-rail dynamic measurement^11,12^. In the study of shoegear-rail system dynamic performance, many scholars established the coupling dynamic model of electric shoegear-conductor rail, and conducted certain exploration on the coupling vibration problem of the power supply of shoegear-rail system^13,14^, and some scholars conducted certain experiments^15^. Simarro^16^ studied the impact of initial contact force on pantograph. Although they have conducted certain researches on the dynamic characteristics of shoegear-rail system, the running speed is low. The vibration of shoegear-rail system will change with initial contact force. A detailed understanding of the vibration of shoegear-rail system under different initial contact forces can help to determine appropriate initial contact force in the production and installation of shoegear-rail system, so as to improve the running performance of trains and reduce the occurrence of accidents, which has important practical significance.

Under a high running speed condition, shoegear-rail coupling vibration is violent. The difficulties encountered in the current research are that the dynamic performance is still absent under the condition. Thus, it is almost impossible to detailedly understand the effects of initial contact force on shoegear-rail coupling vibration under a high speed condition. However, it is very necessary to inveatigate shoegear-rail coupling vibration characteristics at high speeds, which can ensure the stability of trains during high-speed operation. Therefore, this work mainly focuses on shoegear-rail system and establishes a coupling model. Then, the numerical simulation of shoegear-rail coupling vibration under different initial contact forces is performed, and the maximum vibration displacement, bending moment, shear force value of the conductor rail, as well as the contact and viscous forces of the shoegear are obtained.

## Mathematical model

### Conductor rail model

**Figure 1:**
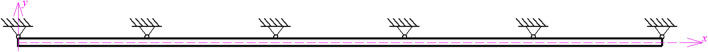
The model of a single conductor rail.

In order to analyze the vibration of shoegear-rail system under different contact forces, the shoegear sliding over a conductor rail is selected here. The conductor rail is simplified into a multi-span continuous Euler–Bernoulli beam with equal section and simple supports, and the fixed position between conductor rail and railway foundation is simplified into a fixed hinged support^17^. The model of a single conductor rail is shown in Fig. 1.

**Figure 2:**
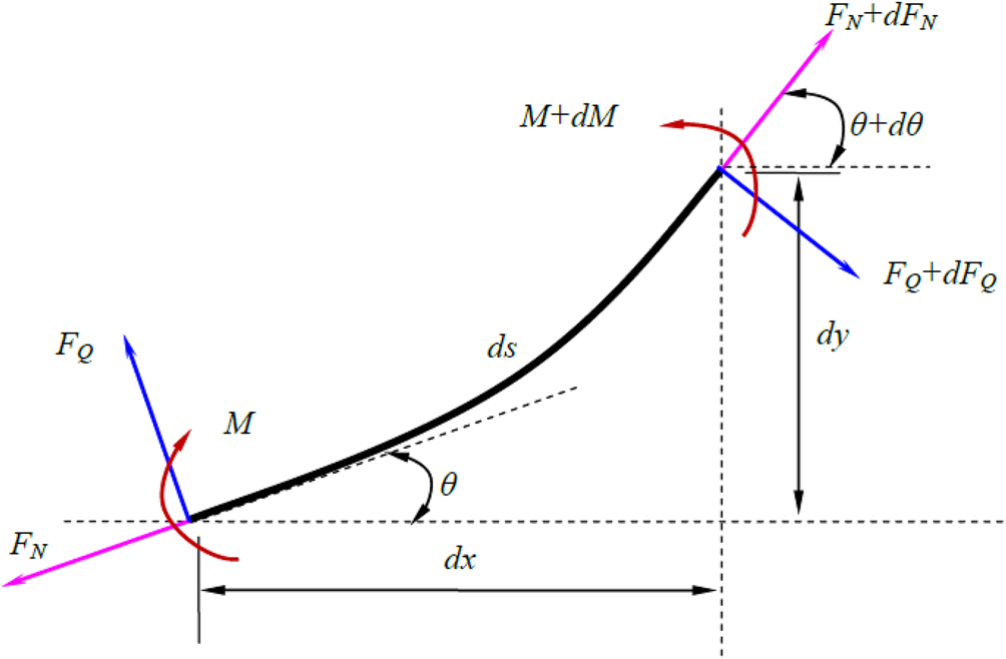
Force diagram of micro-element conductor rail.

In order to obtain the vibration equation of the conductor rail, a segment of the micro-element conductor rail is selected for mechanical analysis, as shown in Fig. 2.

Where, $F_{Q}$ and $F_{N}$ represent the shear and axial forces, $\theta$ is the angle, $M$ is the bending moment, $s$ is the arc coordinate, $x$ and $y$ are the rectangular coordinates.

According to mechanics of materials, we can know

1 $$ \frac{d\theta }{{ds}} = \frac{1}{\rho } = \frac{M}{EI} $$

Namely:

2 $$ M = EI\frac{d\theta }{{ds}} $$

where $EI$ is the bending stiffness.

The relationship between bending moment and shear force is as follows:

3 $$ F_{Q} = \frac{dM}{{ds}} $$

The vibration in the $x$ direction is analyzed.

4 $$ a_{x} = \frac{{d^{2} x}}{{dt^{2} }} = \frac{{dF_{x} }}{dm} = \frac{{dF_{x} }}{{\rho_{s} ds}} $$

where, $a_{x}$ represents the vibration acceleration in the $x$ direction, $dF_{x}$ is the resultant force in the $x$ direction, $dm$ is the mass of the micro-element conductor rail, $\rho_{s}$ is the linear density (Multiplying density $\rho$ and cross-sectional area $A$), and $ds$ is the length. The resultant force in the $x$ direction is calculated as

5 $$ dF_{x} = \left( {F_{Q} + dF_{Q} } \right)\sin \left( {\theta + d\theta } \right) - F_{Q} \sin \theta + \left( {F_{N} + dF_{N} } \right)\cos \left( {\theta + d\theta } \right) - F_{N} \cos \theta $$

Similarly, analyzing the vibration in the $y$ direction, there is

6 $$ a_{y} = \frac{{d^{2} y}}{{dt^{2} }} = \frac{{dF_{y} }}{dm} = \frac{{dF_{y} }}{{\rho_{s} ds}} $$

where, $a_{y}$ is the vibration acceleration in the $y$ direction, and $dF_{y}$ is the resultant force in the $y$ direction, which can be obtained as

7 $$ dF_{y} = - \left( {F_{Q} + dF_{Q} } \right)\cos \left( {\theta + d\theta } \right) + F_{Q} \cos \theta + \left( {F_{N} + dF_{N} } \right)\sin \left( {\theta + d\theta } \right) - F_{N} \sin \theta + F_{C} $$

where, $F_{C}$ represents the contact force generated by the interaction between the conductor rail and the electric shoegear.

### Electric shoegear model

**Figure 3:**
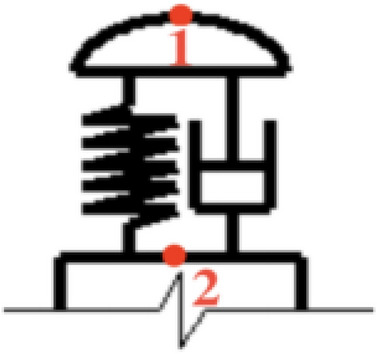
Equivalent model of electric shoegear.

The electric shoegear is mainly composed of slipper, swing arm, spring, and base. It is actually a system including inertia, mass, elastic stiffness and damping. Based on the principle of virtual displacement, the mass block equivalent model of electric shoegear can be derived^18^. Therefore, the electric shoegear can be equivalent to a spring-damping oscillator with a single degree of freedom, which is established as a mass block model and simplified into a mass-spring-damping combination component, as shown in Fig. 3. In order to analyze the vibration, two specific points (1 and 2) on the electric shoegear are selected.

The vibration equation of electric shoegear is:

8 $$ a_{{y_{1} }} = \frac{{d^{2} y_{1} }}{{dt^{2} }} = \frac{{F_{spring} + F_{viscous} - F_{C} }}{m} $$

where, $y_{1}$ represents the $y$ coordinate of point $1$, $F_{spring}$ is the elastic force, and $F_{viscous}$ is the viscous resistance.

The elastic force is calculated as

9 $$ F_{spring} = K_{s} \left[ {l_{12} - \left( {y_{1} - y_{2} } \right)} \right] $$

where, $K_{s}$ represents the elastic coefficient of the spring, $l_{12}$ is the distance between points $1$ and $2$ in the natural state (when the force of the spring is $0$), $y_{2}$ represents the $y$ coordinate of point $2$. The $y_{2}$ value remains constant when the electric shoegear slides on the surface of the conductor rail. The viscous resistance can be obtained as.

10 $$ F_{viscous} = - \eta v_{1} = - \eta \frac{{dy_{1} }}{dt} $$

where, $\eta$ is the viscosity and $v_{1}$ is the vibration velocity in the $y$ direction.

### Shoegear-rail coupling model

**Figure 4:**
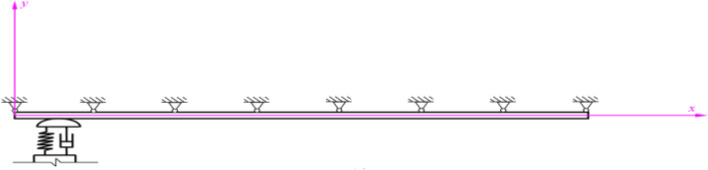
Shoegear-rail coupling model.

The conductor rail is fixed and the electric shoegear slides on the surface of the conductor rail with the operation of train. They interact with each other due to their contact and there is complex coupling vibration. The contact force changes with time. The shoegear-rail coupling model is shown in Fig. 4. A value of two meters for the spacing between these fixed hinge supports is taken in the model.

In order to describe the coupling force between shoegear and rail, the penalty function method^10^ is adopted. Equation (11) can be used to calculate the force.

11 $$ \begin{gathered} \begin{array}{*{20}c} {When} & {\begin{array}{*{20}c} {y_{1} \ge y_{R} ,} & {} \\ \end{array} } & {} \\ \end{array} F_{C} = K_{C} \left( {y_{1} - y_{R} } \right) \hfill \\ \hfill \\ \begin{array}{*{20}c} {When} & {\begin{array}{*{20}c} {y_{1} < y_{R} ,} & {} \\ \end{array} } & {} \\ \end{array} F_{C} = 0 \hfill \\ \end{gathered} $$

where, $K_{C}$ is the contact stiffness between the electric shoegear and the conductor rail, $y_{R}$ is the displacement of the $y$ direction along the neutral axis of the conductor rail.

## Numerical solution

### Calculation method

**Figure 5:**
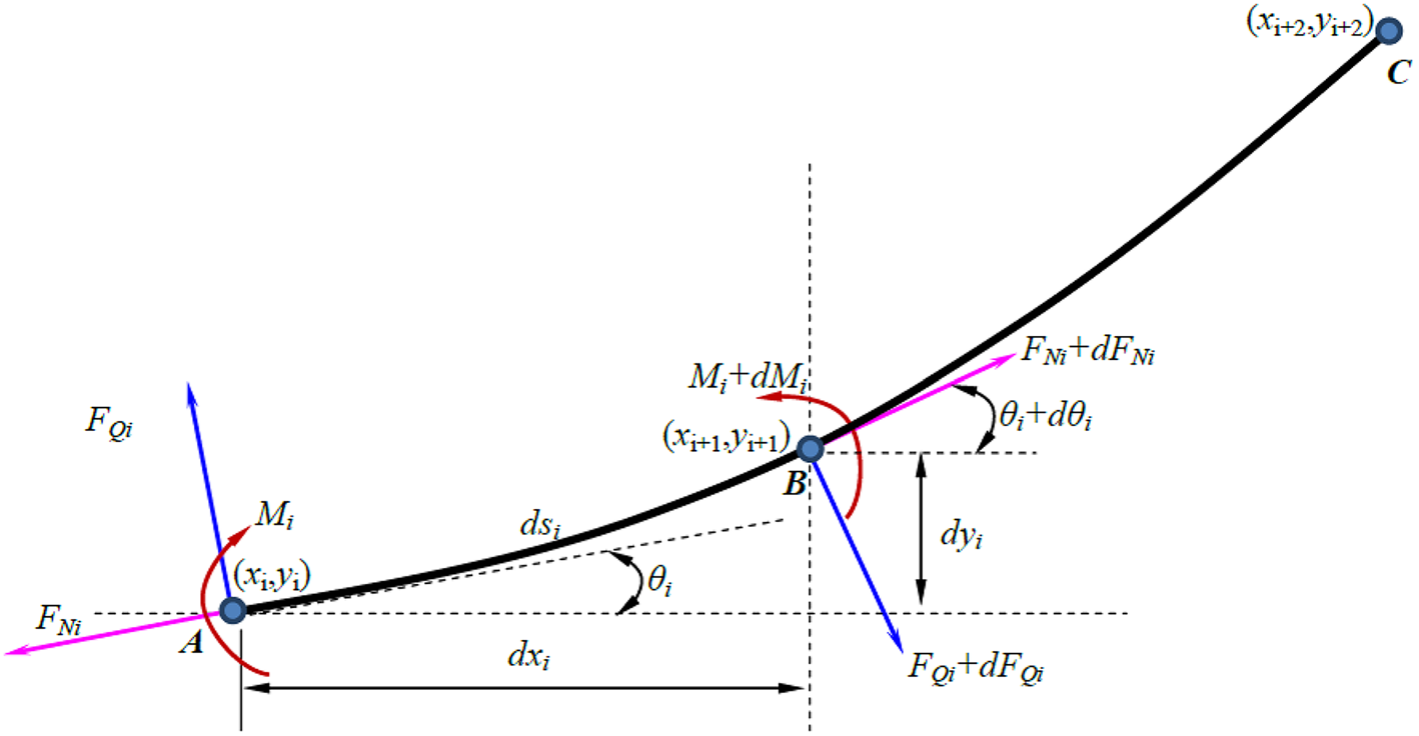
The map of numerical solution analysis.

The vibration equation is a group of differential equations and it is very difficult to obtain the analytical solution. Therefore, a numerical method is used to solve the vibration equation. Figure 5 depicts the numerical solution analysis.

The coordinates of points $A$,$B$ and $C$ are $\left( {x_{i} ,y_{i} } \right)$, $\left( {x_{i + 1} ,y_{i + 1} } \right)$ and $\left( {x_{i + 2} ,y_{i + 2} } \right)$ respectively. The angles, bending moments, shear forces and axial forces of the beam segment at these three points are $\theta_{i}$, $M_{i}$, $F_{Qi}$, $F_{Ni}$, $\theta_{i + 1}$, $M_{i + 1}$, $F_{Qi + 1}$, $F_{Ni + 1}$ and $\theta_{i + 2}$, $M_{i + 2}$, $F_{Qi + 2}$_,_ *F*_*Ni*+2_ respectively. The relationship among angle, bending moment, shear force and axial force is as follows:

12 $$ \theta_{i + 1} = \theta_{1} + d\theta_{i} ,M_{i + 1} = M_{i} + dM_{i} ,F_{Qi + 1} = F_{Qi} + dF_{Qi} ,F_{Ni} + dF_{Ni} \,\theta_{i = 2} = \theta_{i + 1} = d\theta_{i + 1} ,M_{i = 2} = M_{i = 1} + dM_{i + 1} ,F_{Qi + 2} = F_{Qi + 1} + dF_{Qi + 1} ,F_{Ni + 2} = F_{Ni + 1} + dF_{Ni + 1} $$

Taking points $A$,$B$ and $C$ on the element segment as examples, the angle, bending moment, shear force and axial force of the continuous beam can be calculated according to the following equations.

13 $$ \theta_{i} = \arctan \left( {\frac{{y_{i + 1} - y_{i} }}{{x_{i + 1} - x_{i} }}} \right) $$

14 $$ \theta_{i + 1} = \theta_{i} + d\theta_{i} = \arctan \left( {\frac{{y_{i + 2} - y_{i + 1} }}{{x_{i + 2} - x_{i + 1} }}} \right) $$

15 $$ d\theta_{i} = \theta_{i + 1} - \theta_{i} = \arctan \left( {\frac{{y_{i + 2} - y_{i + 1} }}{{x_{i + 2} - x_{i + 1} }}} \right) - \arctan \left( {\frac{{y_{i + 1} - y_{i} }}{{x_{i + 1} - x_{i} }}} \right) $$

16 $$ M_{i + 1} = EI\frac{{d\theta_{i} }}{ds} = \frac{{EI\left[ {\arctan \left( {\frac{{y_{i + 2} - y_{i + 1} }}{{x_{i + 2} - x_{i + 1} }}} \right) - \arctan \left( {\frac{{y_{i + 1} - y_{i} }}{{x_{i + 1} - x_{i} }}} \right)} \right]}}{ds} $$

17 $$ F_{Qi + 1} = \frac{{dM_{i} }}{ds} = \frac{{M_{i + 1} - M_{i} }}{ds} $$

18 $$ F_{Ni} = EA\frac{{\sqrt {dx_{i}^{2} + dy_{i}^{2} } - ds}}{ds} = EA\frac{{\sqrt {\left( {x_{i + 1} - x_{i} } \right)^{2} + \left( {y_{i + 1} - y_{i} } \right)^{2} } - ds}}{ds} $$

In the program calculation, we use the finite difference method for numerical integration, discretizing the differential equation into a difference equation, and then solving the numerical solution on the scattered point. Using the above numerical method and writing a MATLAB program, we can find out the relationship among the displacement, velocity, bending moment and axial force of the transverse and longitudinal vibrations of the conductor rail, and then get the maximum stress and strain at the cross section of the conductor rail in the vibration process.

For the selection of time step, we adopt an adaptive time step strategy. During the calculation process, the time step is automatically adjusted based on the changes in the solution to ensure the accuracy and stability of the calculation. Specifically, if the change in the solution is small, the time step is appropriately reduced to improve accuracy. If the solution changes significantly, the time step is appropriately increased to maintain the stability of the calculation. After many tests, the element length is set to be 5 mm and the calculation accuracy can be assured.

### Parameter determination

**Figure 6:**
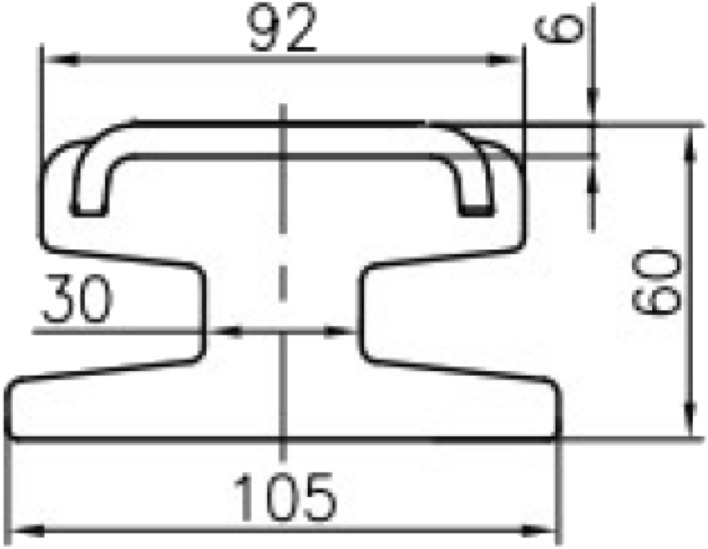
Actual cross section of conductor rail.

**Figure 7:**
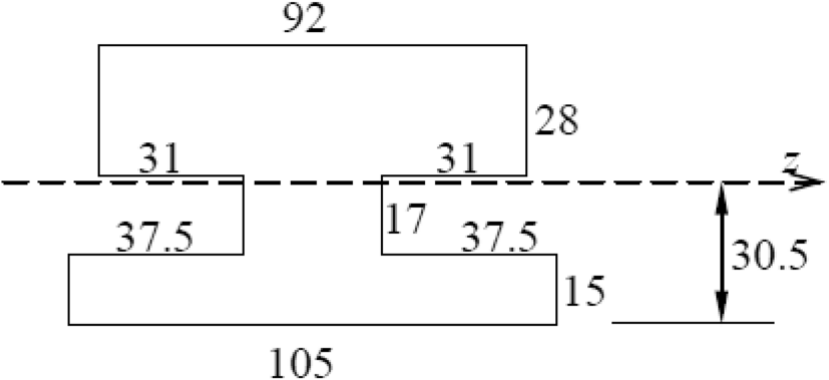
Simplified cross section of conductor rail.

The cross section shape of the conductor rail is rather complex, as shown in Fig. 6. We simplify it in Fig. 7. The units in Figs.6,7 are mm.

The conductor rail is made of steel and aluminum. Its contact surface and bracket are respectively made of steel strip and aluminum alloy. After simplifying the cross section of the conductor rail, the required parameters can be obtained, as shown in Tables.1 and 2.

**Table 1:** Parameters of the contact rail.

Elastic modulus (GPa)	bending stiffness $\left( {Pa \cdot m^{4} } \right)$	Cross-sectional Area $\left( {m^{2} } \right)$	Density $\left( {kg \cdot m^{ - 3} } \right)$	Single contact rail length $\left( m \right)$	Bending section coefficient $\left( {m^{3} } \right)$
70	$1.181 \times 10^{5}$	$4.236 \times 10^{ - 3}$	$7.83 \times 10^{3}$	$50$	$5.5317 \times 10^{ - 5}$

**Table 2:** Parameters of the electric shoegear.

Quality $\left( {kg} \right)$	Elastic coefficient $\left( {N \cdot m^{ - 1} } \right)$	Viscosity ($N \cdot s \cdot m^{ - 1}$)
$3$	$1.04 \times 10^{4}$	$10$

Here, we set the contact stiffness of the shoegear-rail system to be $2 \times 10^{4} N \cdot m^{ - 1}$.

## Results

The vibration of the shoegear-rail system varies with the train speed. In order to study the vibration under different initial contact forces, the running speed of the train is set to be $300km/h$. In order to conduct dynamic characteristic analysis, we need to set a reasonable range of initial contact forces. This scope should be large enough to cover possible changes, but not too broad and lose analytical value. We refer to the shoegear-rail contact force data in actual high-speed train power supply systems. Through on-site measurements and data collection, we adopt an initial contact force distribution range of 70–160 N for our analysis. When other parameters are the same, the initial contact force is selected as 70, 100, 130 and 160 N respectively, and the corresponding results are calculated as follows.

**Figure 8:**
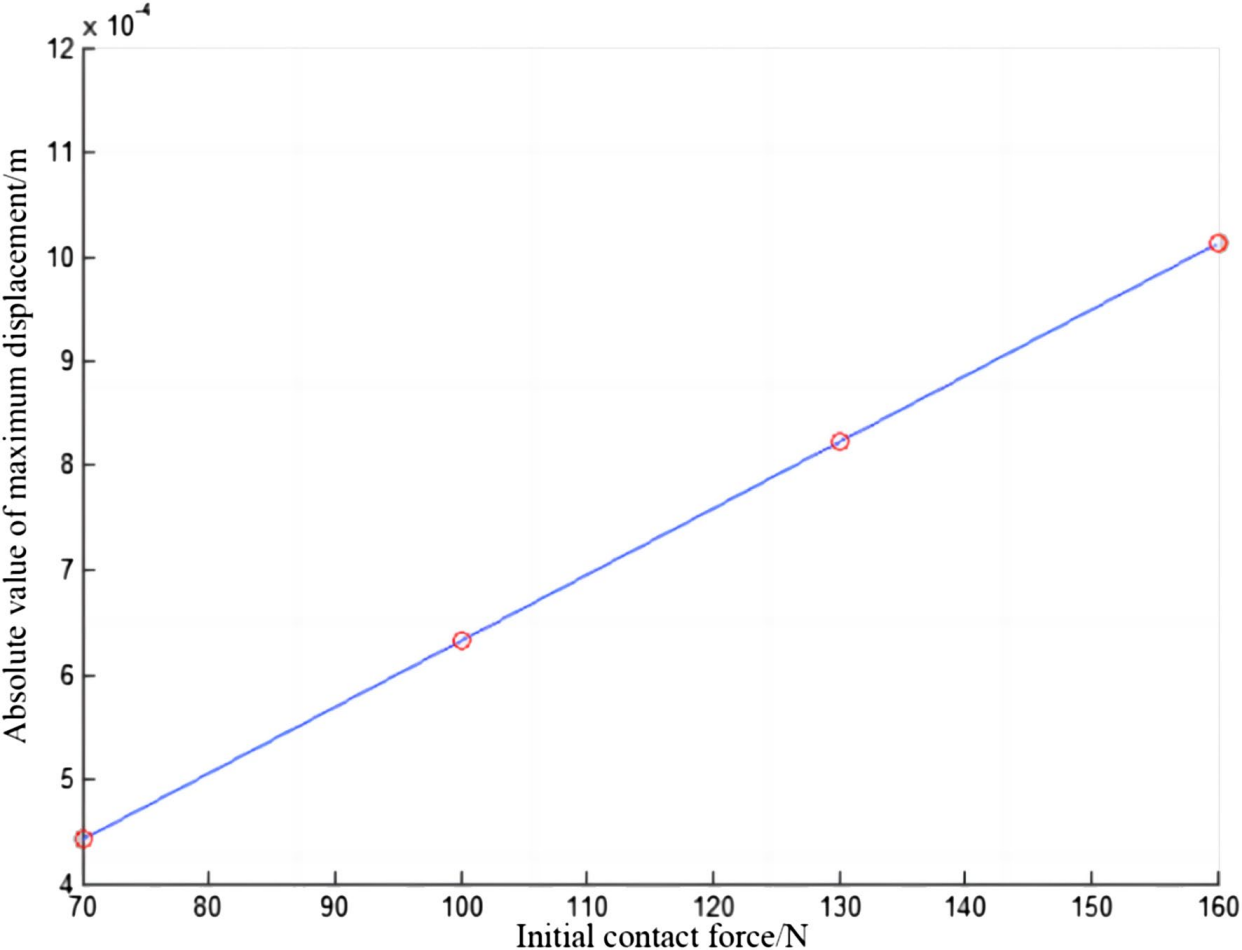
Absolute value of maximum displacement of conductor rail.

The change of the absolute value of the maximum vibration displacement of the conductor rail with the initial contact force is shown in Fig. 8. The absolute value of the maximum vibration displacement increases with the increase of the initial contact force, and they are positively correlated and the relationship is approximately linear. However, even if the initial contact force is 160 N, the absolute value of maximum displacement of the conductor rail is small, roughly 1 mm.

**Figure 9:**
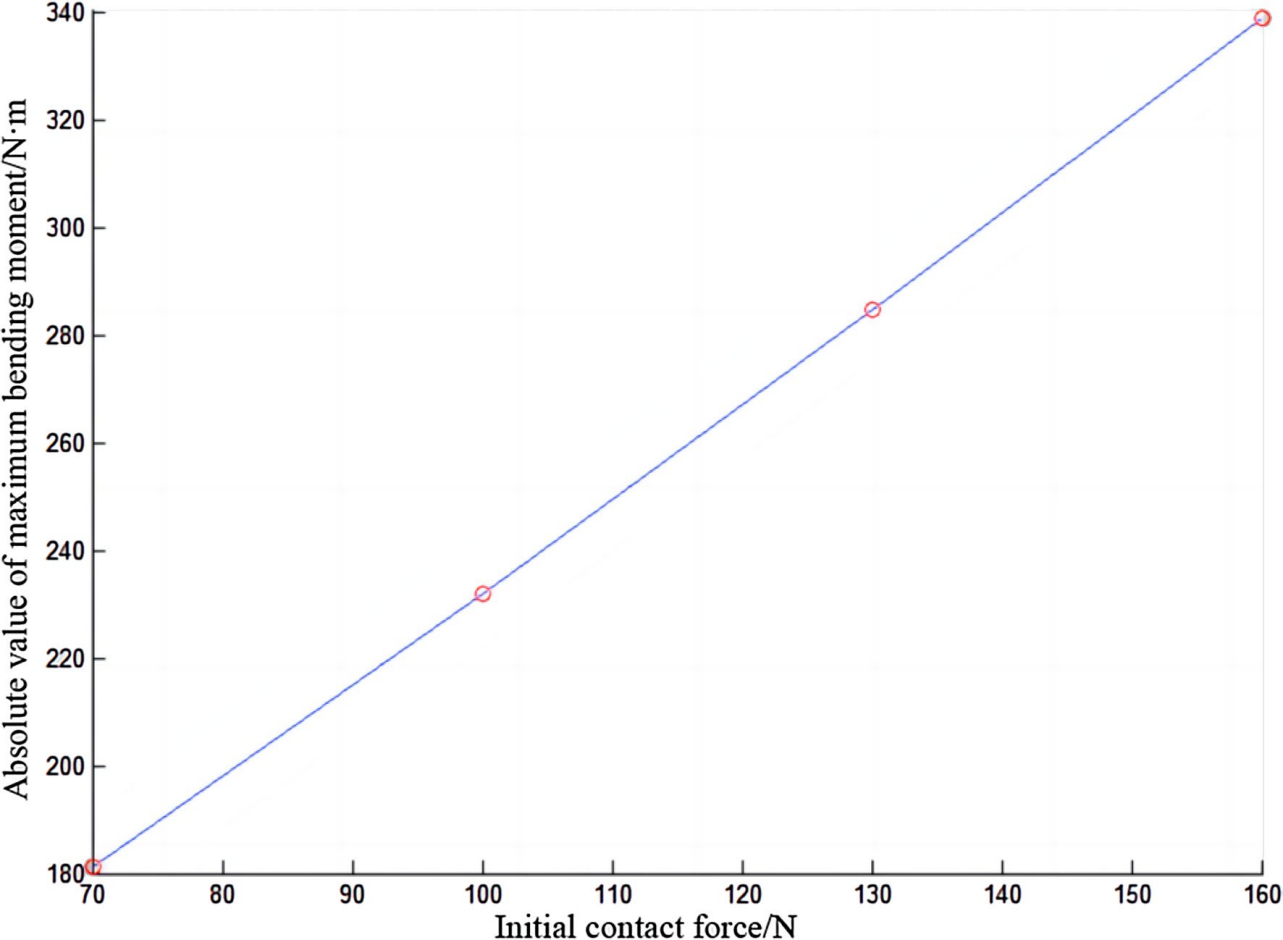
Absolute value of maximum bending moment of conductor rail.

In Fig. 9, the absolute value of the maximum bending moment of the conductor rail increases with the increase of the initial contact force. The line formed by the points in the figure is almost a straight line, which also means that the absolute value of the maximum bending moment of the conductor rail has a linear relationship with the initial contact force.

**Figure 10:**
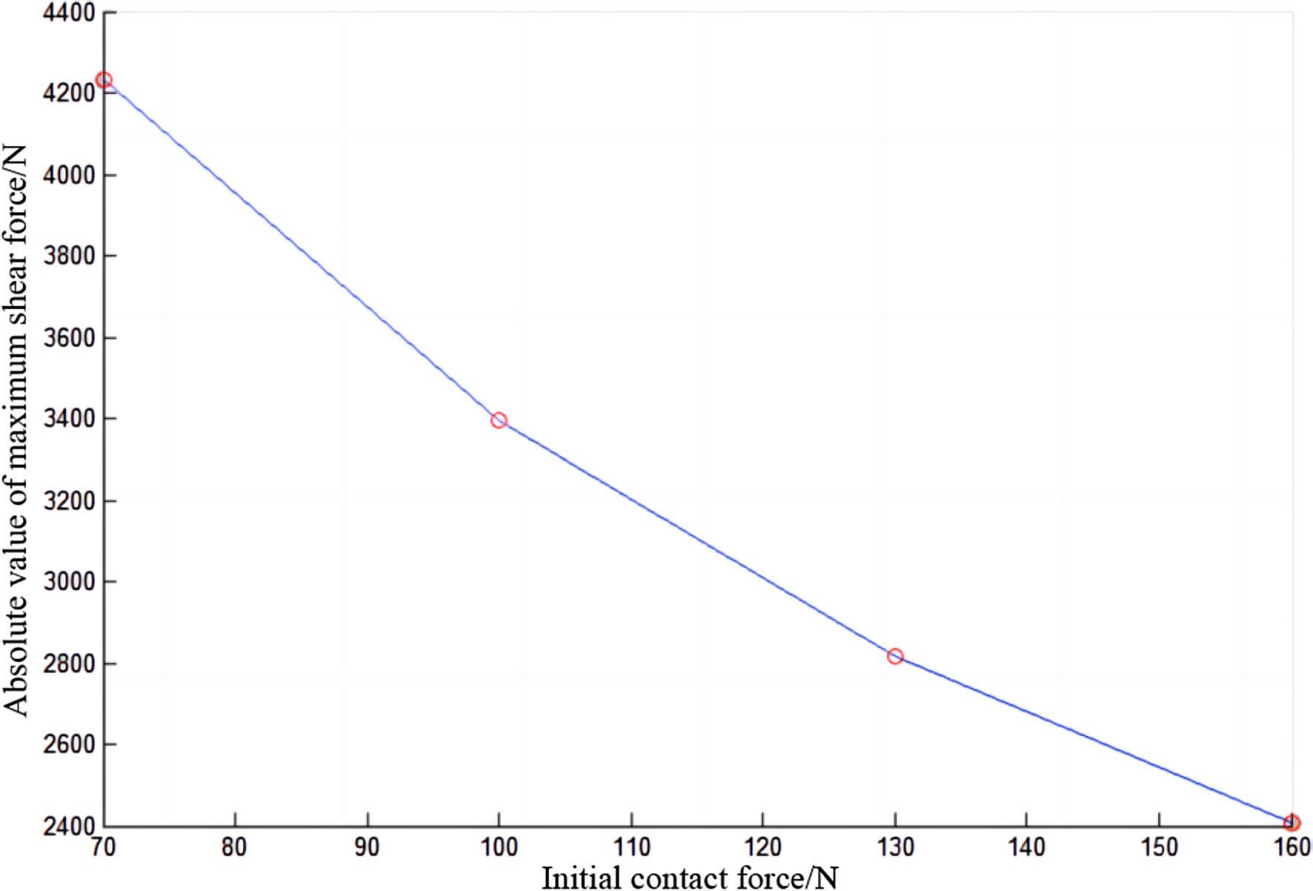
Absolute value of maximum shear force of conductor rail.

In Fig. 10, the absolute value of the maximum shear force of the conductor rail decreases with the increase of the initial contact force, which indicates that the absolute value of the maximum shear force is negatively correlated with the initial contact force. The relationship is not linear.

**Figure 11:**
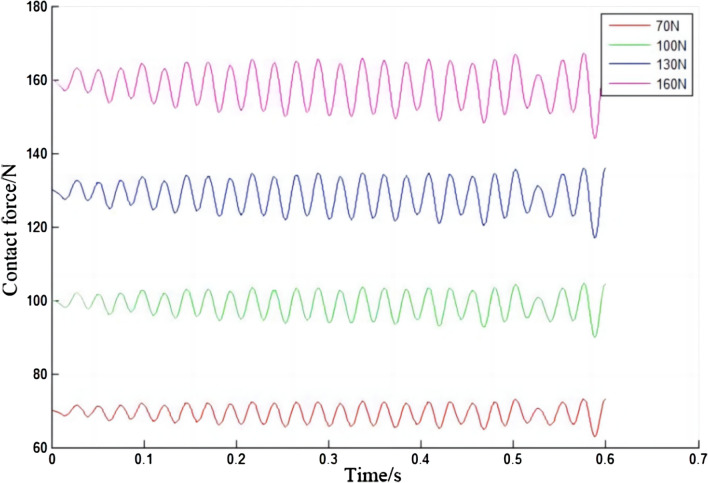
Contact force of electric shoegear.

**Figure 12:**
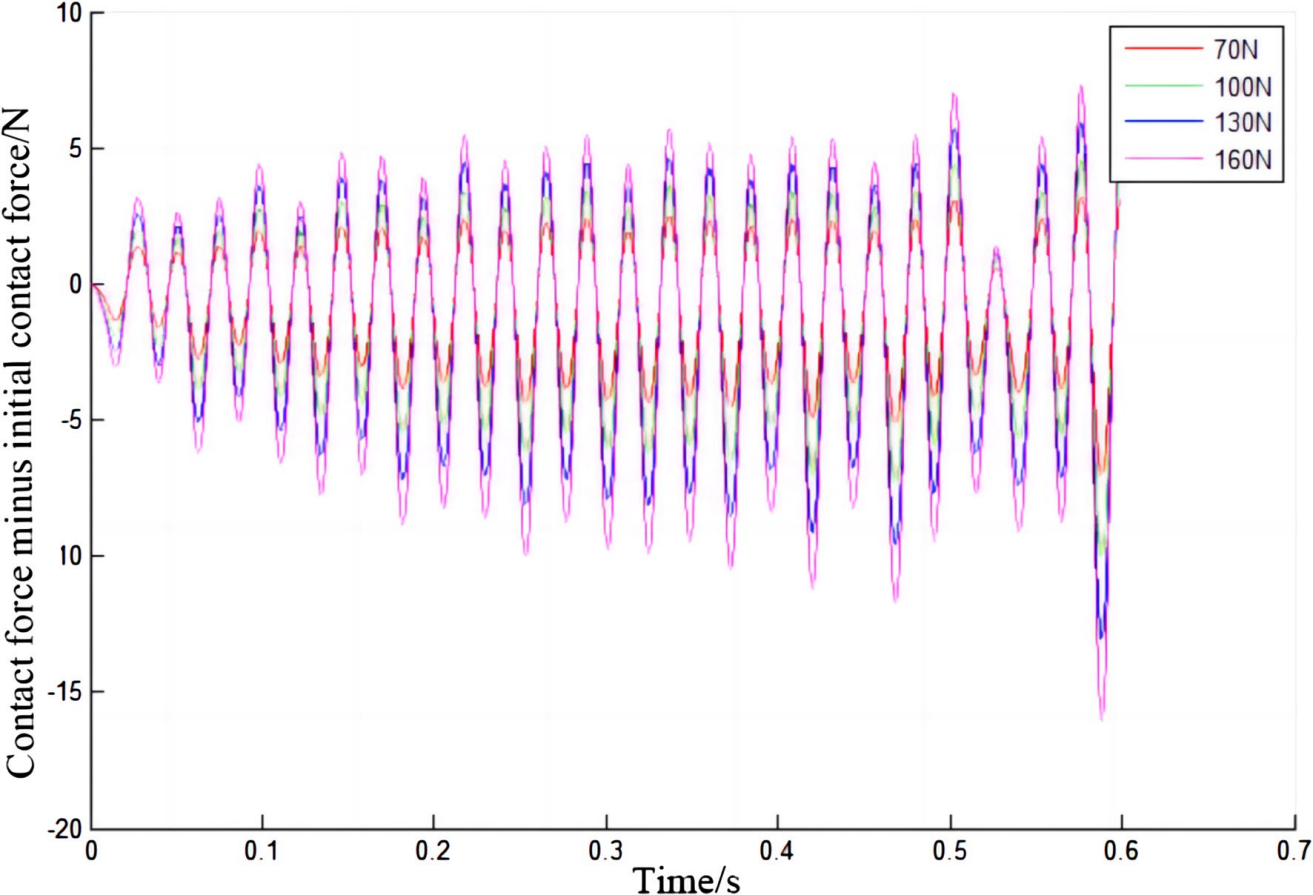
Contact force increments of electric shoegear.

**Figure13:**
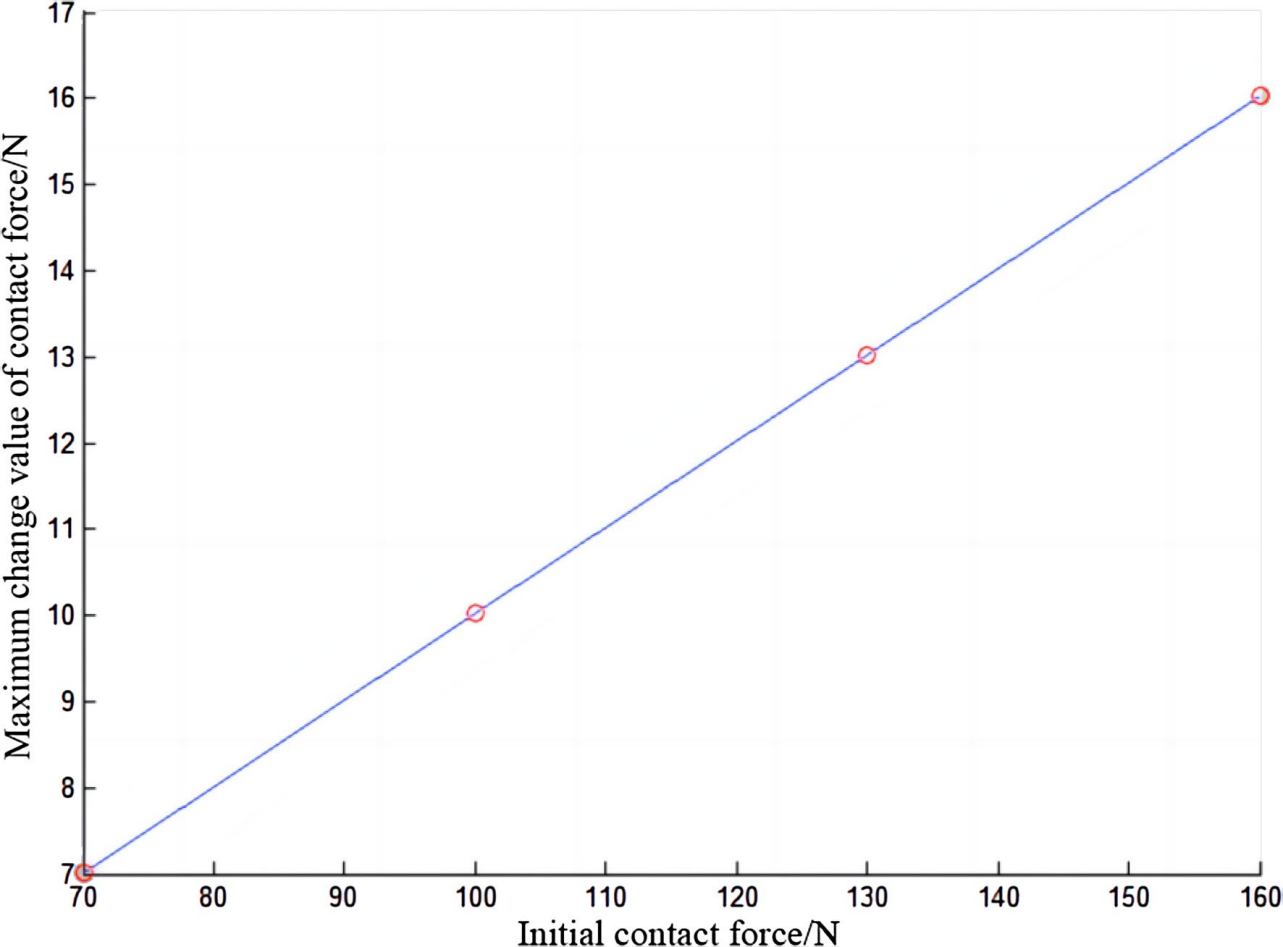
Maximum change in contact force of electric shoegear.

Under different initial contact forces, the force between the electric shoegear and the conductor rail will also be different, as shown in Fig. 11. As the initial contact force increases, the contact force of electric shoegear will also increase. During the train operation, the contact force of electric shoegear changes periodically, sometimes greater than the initial contact force, and sometimes less than the initial contact force. Moreover, with the increase of the initial contact force, the vibration frequency does not change significantly, while the vibration amplitude gradually increases. It can be seen in Fig. 11, but it is even more evident in Fig. 12. In Fig. 12, it can be seen that the amplitude tends to increase with time. From Fig. 12, it is obvious that the vibration frequency does not change with the difference of the initial contact force. The maximum variation of the contact force of the electric shoegear under different initial contact forces is summarized, and the relationship diagram between the maximum variation of the contact force of the electric shoegear and the initial contact force is made, as shown in Fig. 13. It can be seen that the maximum variation of the contact force is positively correlated with the initial contact force and has a linear relationship.

**Figure 14:**
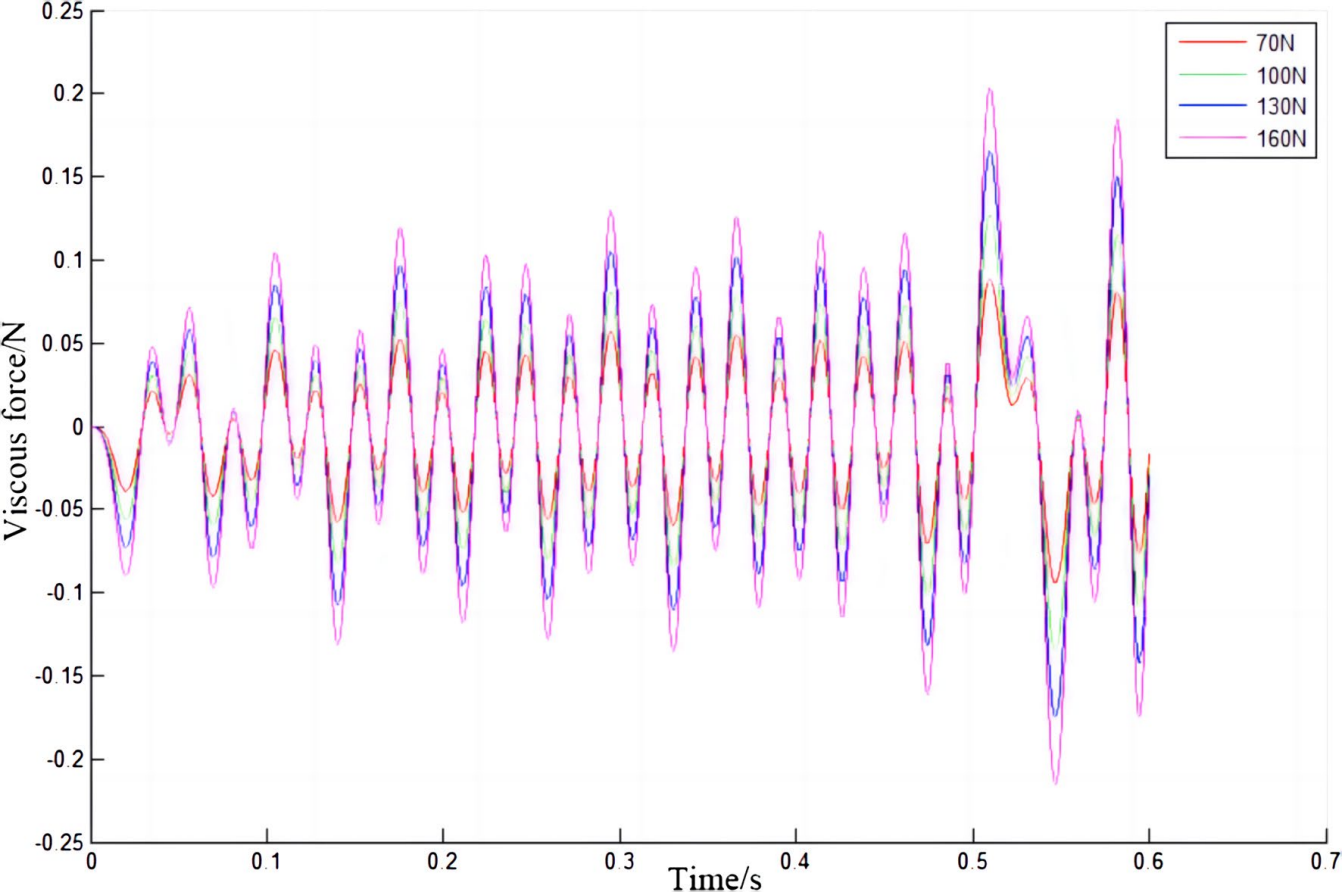
Viscous force of electric shoegear.

**Figure 15:**
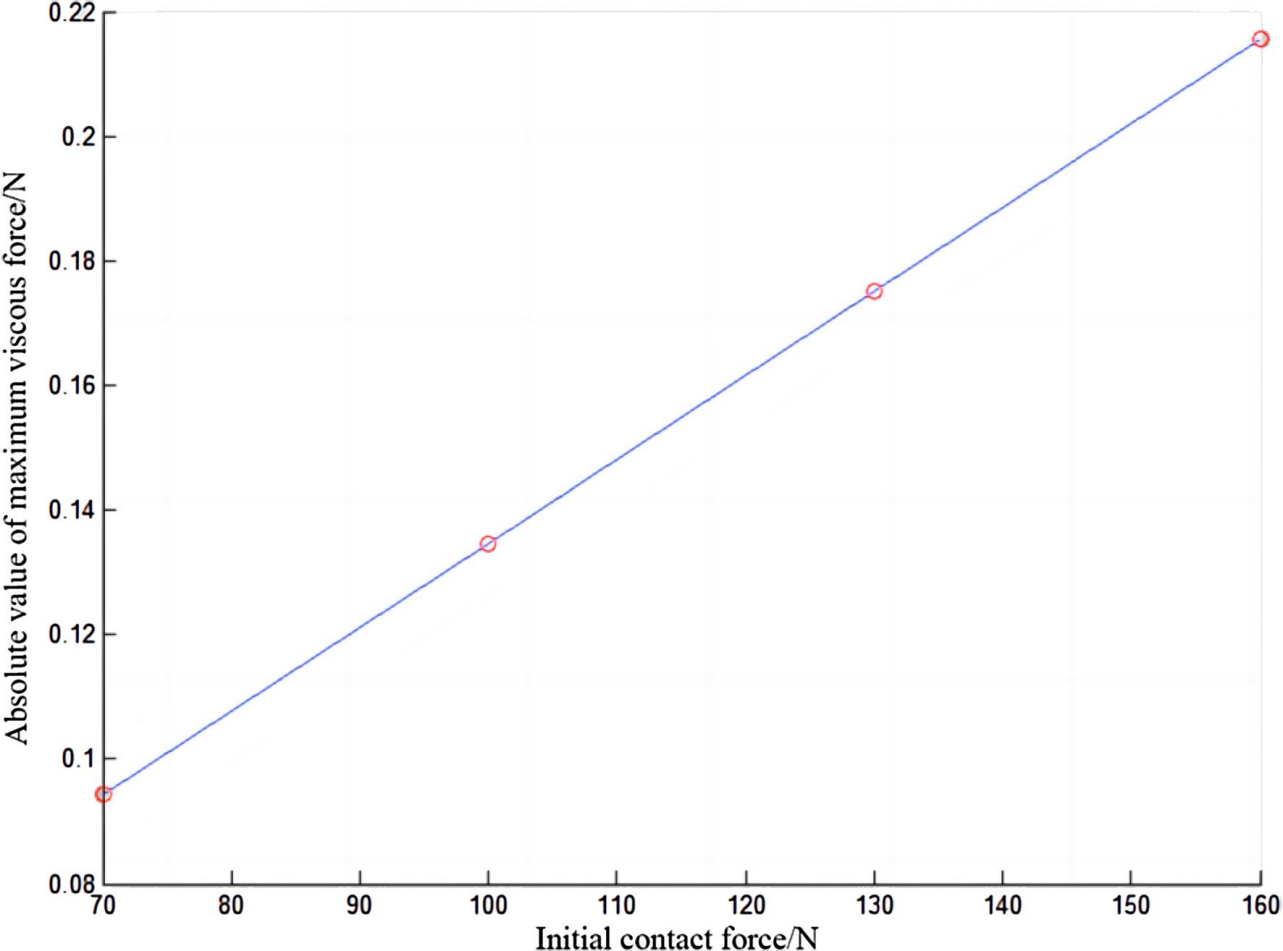
Absolute value of maximum viscous force of electric shoegear.

The viscous force of the electric shoegear will change periodically, as shown in Fig. 14. With the increase of the initial contact force, the change of the viscous force of electric shoegear becomes more and more drastic, and the amplitude increases. As shown in Fig. 15, when the relationship diagram between the absolute value of the maximum viscous force of the electric shoegear and the initial contact force is made, it is found that the absolute value of the maximum viscous force increases with the increase of the initial contact force, and the relationship is linear.

**Figure 16:**
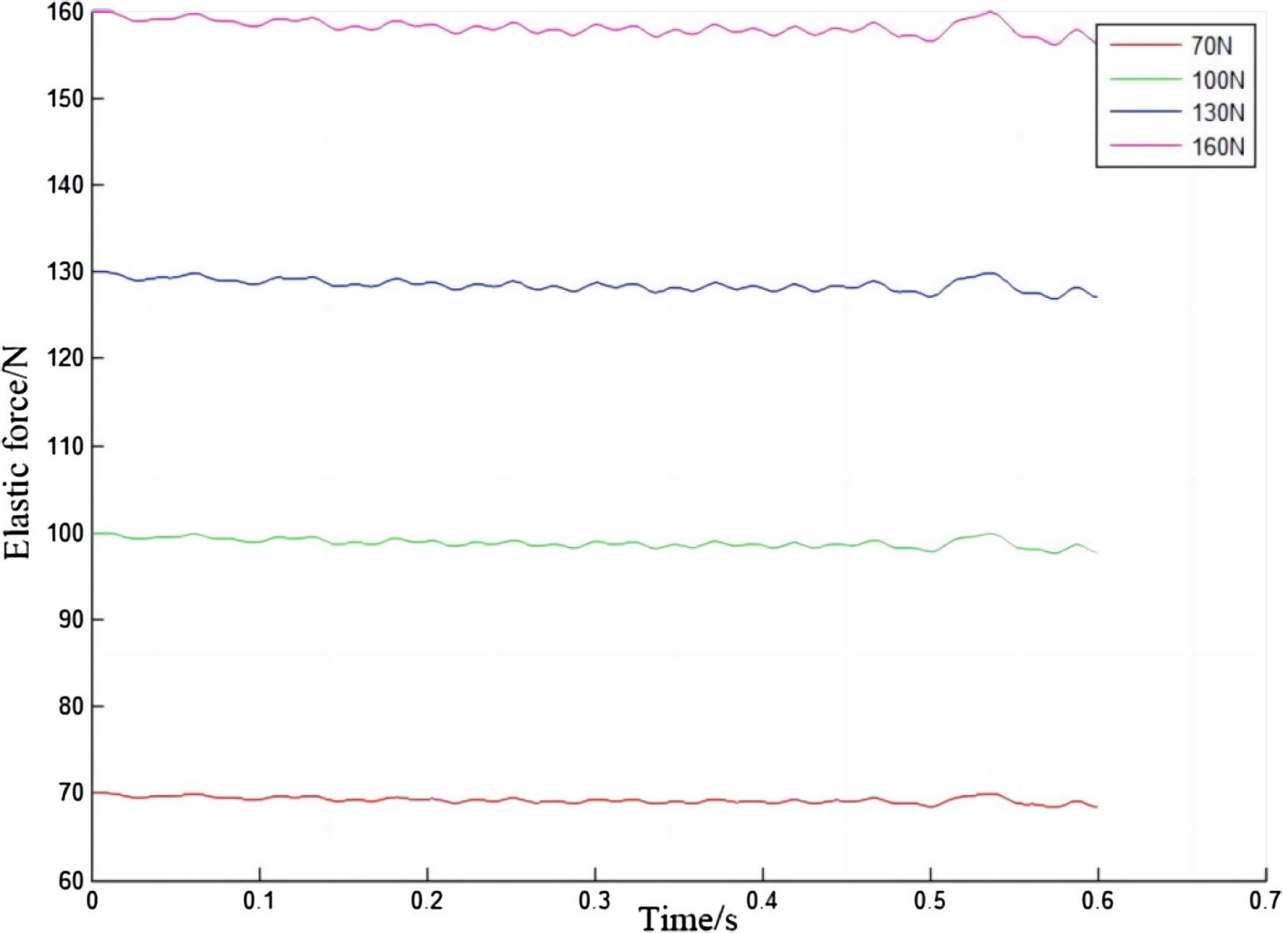
Elastic force of electric shoegear.

**Figure 17:**
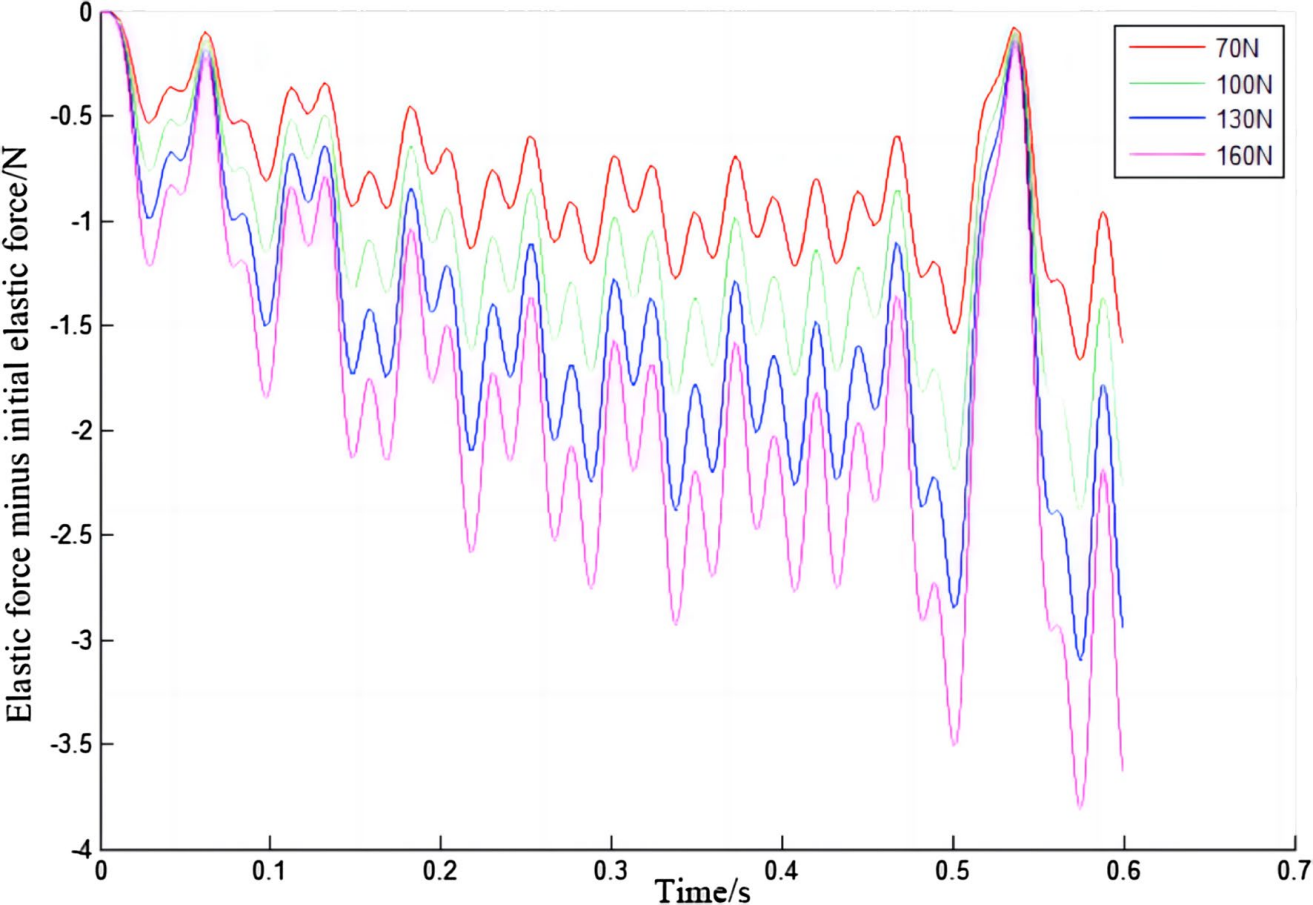
Elastic force increments of electric shoegear.

**Figure 18:**
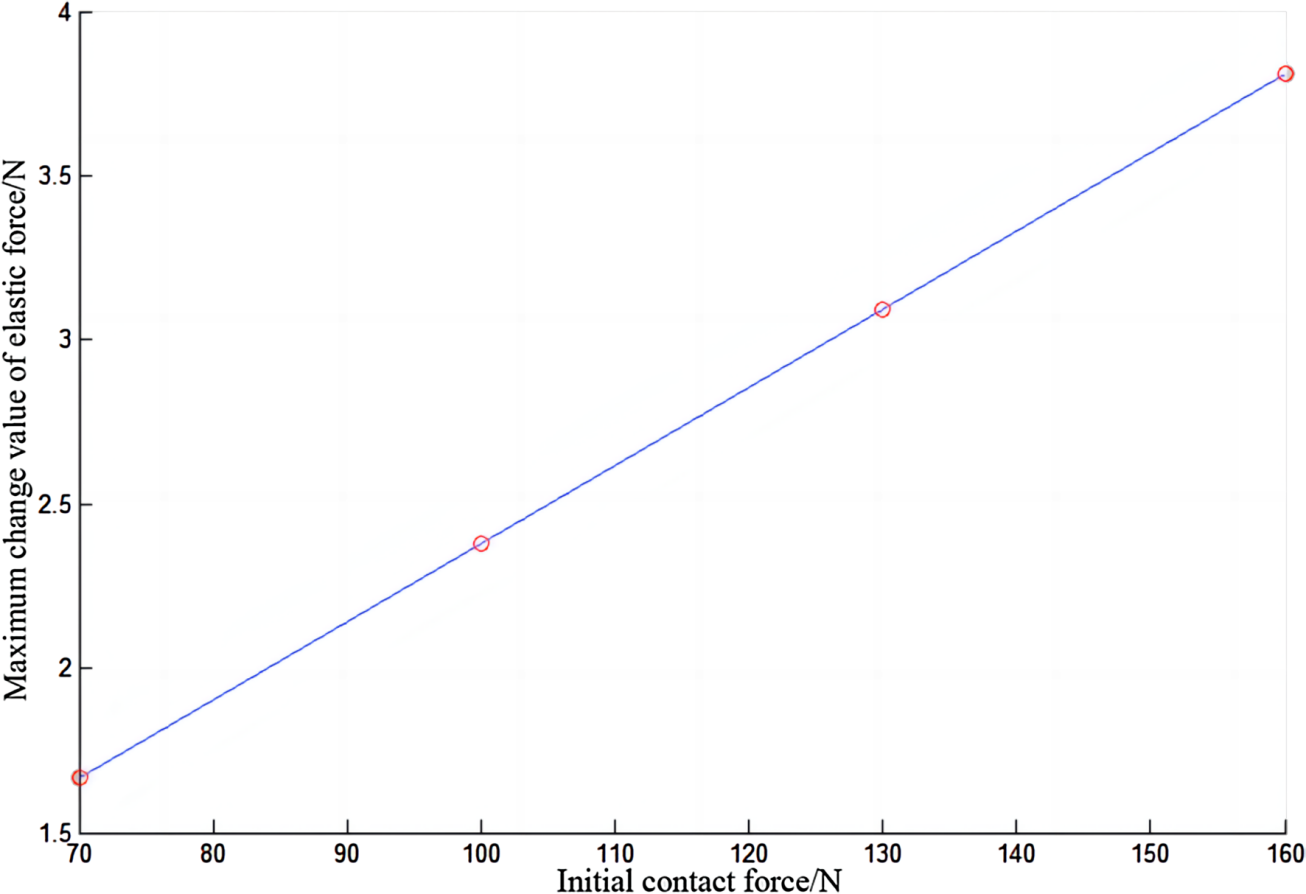
Maximum change in elastic force of electric shoegear.

In Fig. 16, the elastic force of the electric shoegear changes periodically with time, sometimes increasing and sometimes decreasing. The greater the initial contact force, the greater the elastic force of the electric shoegear. The relationship between the elastic force increment and the initial contact force is made, as shown in Fig. 17. It is clear that the elastic force increment of the electric shoegear is negative, which means that the elastic force will not exceed the initial elastic force. When the time is about 0.53 s, the elastic force is closest to the initial elastic force. The relationship between the maximum variation of the elastic force and the initial contact force is made, as shown in Fig. 18. It can be found that the maximum variation of the elastic force increases with the increase of initial contact force, and the relationship is linear.

**Figure 19:**
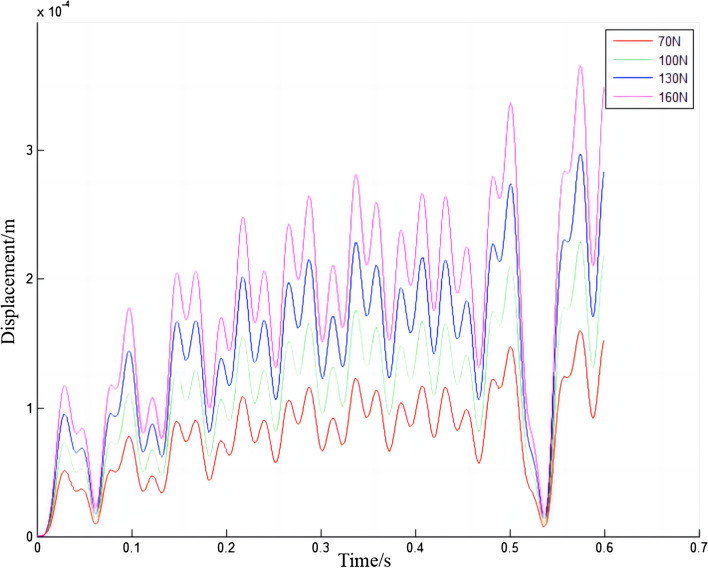
Displacement of electric shoegear.

**Figure 20:**
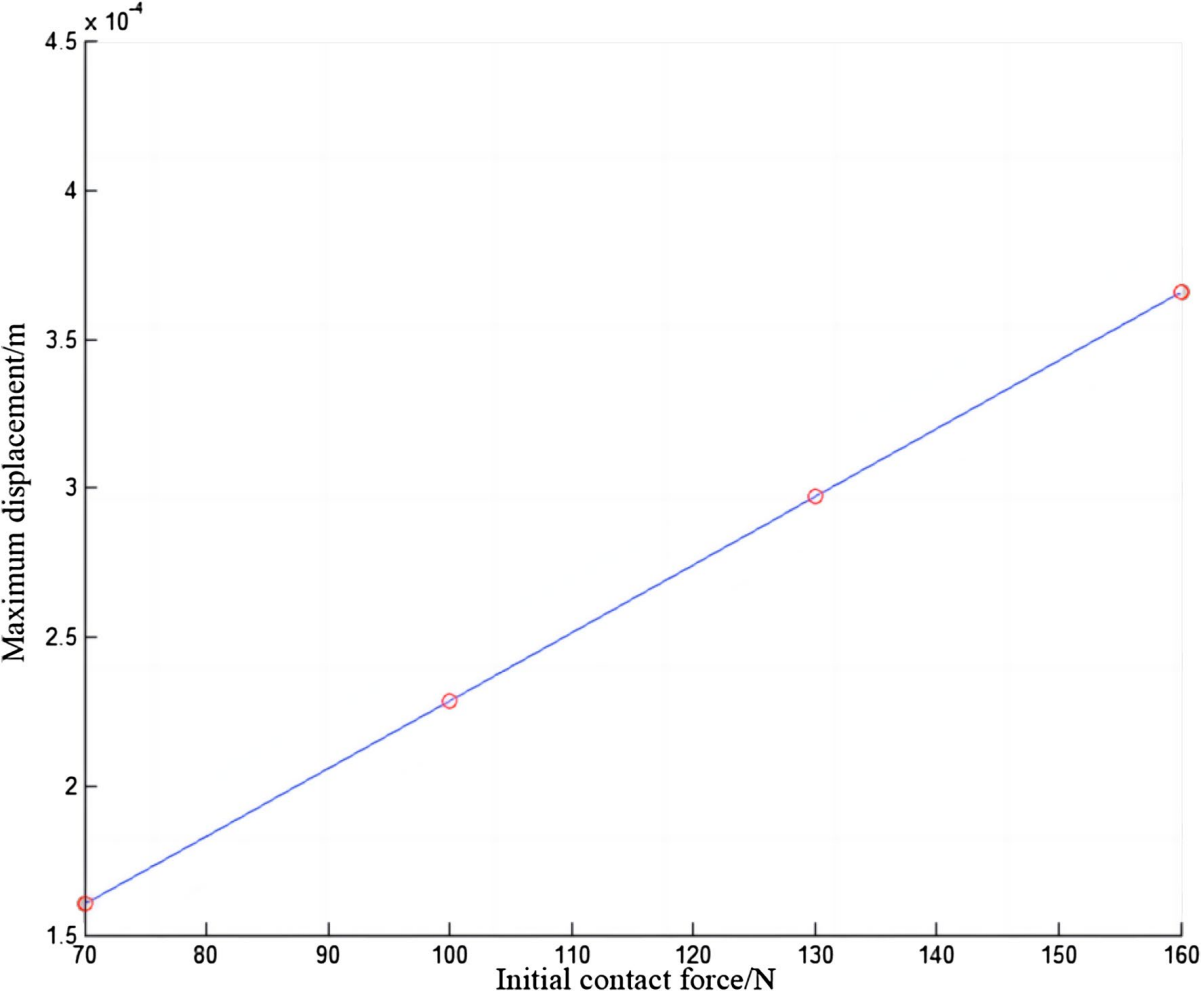
Maximum displacement of electric shoegear.

In Fig. 19, the displacement of the electric shoegear will change repeatedly with time, sometimes increasing, and sometimes decreasing. The displacement of the electric shoegear will increase with the increase of initial contact force. The displacement is always positive, which also indicates that the position of the electric shoegear will not fall below the initial position during the train operation. Around 0.53 s, the displacement of the electric shoegear is closest to zero. The maximum displacement values of the electric shoegear are summarized and Fig. 20 is made. It can be found that the larger the initial contact force, the higher the maximum displacement value of the electric shoegear, and the relationship is linear.

## Discussion

When the train is moving, the vibration of the electric shoegear-conductor rail system is different due to the difference of the initial contact force. The absolute value of the maximum bending moment and the absolute value of the maximum displacement increase with the increase of the initial contact force in the range of 70–160 N when other parameters remain unchanged. They have a linear relationship with the initial contact force. On the other hand, the absolute value of the maximum shear force of the conductor rail decreases with the gradual increase of the initial contact force, and the absolute value of the maximum shear force is not linear with the initial contact force. When other parameters remain unchanged, as the initial contact force between the electric shoegear and the conductor rail gradually increases, in the range of 70–160 N, it is found that the maximum change in the absolute value of the contact force, the maximum change in the elastic force and the maximum displacement value of the electric shoegear gradually increase, and they are linear with the initial contact force. In addition, the viscous force, elastic force and displacement of the electric shoegear will change periodically, but the initial contact force only affects their amplitudes, and does not affect the frequencies and periods. As the initial contact force increases gradually, the contact force between the electric shoegear and the conductor rail increases gradually. The maximum variation of the contact force also increases gradually and has a linear relationship with the initial contact force. The contact force between the electric shoegear and the conductor rail will also change periodically, and it is the same as the viscous force and elastic force of the electric shoegear. The initial contact force only has an effect on its amplitude, but does not change the frequency and period.

The innovation of the research is to investigate the effects of initial contact force on the dynamic performance of shoegear-rail system under a high speed condition. Although the speed of urban rail transit has not yet reached 300 km/h, this speed is a common speed for high-speed railways and one of the directions for the development of urban rail transit in the future. Therefore, from a research perspective, exploring the shoegear-rail coupling vibration characteristics at this speed has certain foresight and can provide theoretical support for the future development of urban rail transit. Studying the shoegear-rail coupling vibration characteristics at high speeds helps to understand the behavior of the system under extreme conditions, which can provide the guidance for design and ensure the stability of trains during high-speed operation. Setting a specific speed value can facilitate comparison with other studies or practical applications. Although this speed may not be achieved in practical applications, such assumptions can provide a benchmark to evaluate the impact of different factors on shoegear-rail coupling vibration. With the development of urban transportation and people's pursuit of travel efficiency, the demand for high-speed rail transit is gradually increasing. Studying the problem of shoegear-rail coupling vibration at high speeds can provide the guidance for practical engineering and solve possible vibration problems during high-speed operation.

The present numerical model is planar, and only the vertical mechanical behaviors are considered. There are reasons why our numerical models only consider vertical mechanical behavior. In the shoegear-rail coupling vibration problem of high-speed train power supply system, the vertical force is the main influencing factor. This model is applicable when dealing with some basic engineering problems that do not require high precision. For example, this model can provide some reference for basic orbit design, stability analysis, etc. However, for more complex and precise engineering problems, this model may not be able to meet the requirements. At this point, we need to establish more complex models, such as three-dimensional models, to consider more influencing factors, improving the computational accuracy of the model, and combining the model with actual data. This is also the research work we will continue to carry out next.

In our model, contact stiffness is calculated based on a constant value. In the shoegear-rail coupling vibration model, the contact stiffness is usually determined based on theoretical and experimental data. The actual contact stiffness is influenced by various factors, such as the material of the contact surface, surface roughness, contact force, etc. Therefore, the contact stiffness may change with time, environmental and operating conditions. In order to more accurately reflect actual engineering practice, it can be considered to set the contact stiffness as a multi-stage function. For example, different stiffness values or stiffness functions can be considered at different stages (such as initial running in, stable operation, wear, etc.). This can better simulate actual situations, such as the gradual stabilization of contact stiffness during the running in period and the decrease of stiffness during the wear period.

We use constant value of contact stiffness mainly for computational simplification. In the later stage, we will conduct research in this area by combining experimental testing and theoretical analysis. Through experimental testing, actual contact stiffness data can be obtained, and theoretical models can be used to analyze the factors and mechanisms that affect contact stiffness, thereby establishing a more accurate mathematical model.

## Conclusion

The vibration amplitude of the electric shoegear and contact force will increase, with the increase of the initial contact force between the electric shoegear and the conductor rail. The results show that the vibration of the electric shoegear and the conductor rail is more intense. The intensity of vibration is closely related to the initial contact force. However, the maximum shear force value of the conductor rail increases as the initial contact force decreases. It can be seen that the lower the initial shoegear-rail contact force, the smaller the vibration, the better the performance of the shoegear-rail system. Our research shows that the initial contact force has a significant impact on the shoegear-rail coupling vibration. When designing the shoegear-rail system, this factor should be fully considered and an appropriate initial contact force value should be selected. According to our research, a suitable initial contact force range may be between 70–160 N, but this is only a rough guide value, and the specific value still needs to be determined based on the actual application environment and working conditions.

However, it should be noted that the determination of these design values is influenced by various factors, such as track materials, vehicle loads, operating speeds, etc. Therefore, future research needs to consider these factors more comprehensively in order to more accurately determine the optimal design value. At the same time, designers should also fully verify and adjust these suggestions based on specific working conditions when applying them.

## Data Availability

The data used to support the findings of this study are included within the article.
